# Visual analysis of trustworthiness studies: based on the Web of Science database

**DOI:** 10.3389/fpsyg.2024.1351425

**Published:** 2024-05-24

**Authors:** Zhen Zhang, Wenqing Deng, Yuxin Wang, Chunhui Qi

**Affiliations:** ^1^Faculty of Education, Henan Normal University, Xinxiang, China; ^2^Faculty of Education, Henan University, Kaifeng, China

**Keywords:** trustworthiness, visual analysis, mapping knowledge domains, Web of Science, CiteSpace

## Abstract

Trustworthiness is the most significant predictor of trust and has a significant impact on people’s levels of trust. Most trustworthiness–related research is empirical, and while it has a long history, it is challenging for academics to get insights that are applicable to their fields of study and to successfully transfer fragmented results into practice. In order to grasp their dynamic development processes through the mapping of network knowledge graphs, this paper is based on the Web of Science database and uses CiteSpace (6.2.R4) software to compile and visualize the 1,463 publications on trustworthy studies over the past 10 years. This paper aims to provide valuable references to theoretical research and the practice of Trustworthiness. The findings demonstrate that: over the past 10 years, trustworthiness-related research has generally increased in volume; trustworthiness research is concentrated in industrialized Europe and America, with American research findings having a bigger global impact; The University of California System, Harvard University, and Yale University are among the high-production institutions; the leading figures are represented by Alexander Todorov, Marco Brambilla, Bastian Jaeger, and others; the core authors are distinguished university scholars; however, the level of cooperation of the core author needs to be improved. The primary journal for publishing research on trustworthiness is the *Journal of Personality and Social Psychology* and *Biology Letters*. In addition, the study focuses on three distinct domains, involving social perception, facial clues, and artificial intelligence.

## 1 Introduction

Trust is an indispensable component of social life (Kennedy and Schweitzer, 2018; Bai et al., 2019; Zhu et al., 2022; Du et al., 2023) and serves as a lubricant for social integration (Yan and Wu, 2016; Milesi et al., 2023). Interpersonal trust is the cornerstone of social interaction and is crucial for society to function properly (Ścigała et al., 2020). However, the factors that arouse trust in others have predominantly not been investigated (Bellucci et al., 2019; Bennett, 2023). Trust is formed up of two elements, trust intention and trust belief, where trust belief is the perceived trustworthiness of others (Kim et al., 2004), including the ability, benevolence, and integrity of others (Mayer et al., 1995). Trustworthiness is the tendency of a trustee to meet the implicit or explicit positive expectations of others for a particular behavior, which reflects the degree to which a trustee is trustworthy (Levine et al., 2018). Trustworthiness is evaluated as (or lacking) the motive for lying as a proximal antecedent variable of trust (Mayer et al., 1995). It is the most significant predictor of trust (Tomlinson et al., 2020) and is perceived as a social norm (Bicchieri et al., 2011). It is the basis for well–functioning interpersonal relationships and usually affects people’s levels of trust. For instance, Van’t Wout and Sanfey discovered that people with high levels of trustworthiness are more likely to gain their peers’ trust and engage in collaboration than those with low levels (van ‘t Wout and Sanfey, 2008).

We are frequently forced to make trust decisions in relationships but also occasionally face situations involving trust violations. The choice of whether to trust others in interpersonal interactions can be considered a trust decision (Radke et al., 2018). Correctly trusting others can yield enormous rewards, while wrongly trusting others can have serious consequences. The collapse of trust connections frequently results in severe economic, emotional, and social costs for people (Bottom et al., 2002); however, trustworthiness may influence this outcome. The expression “breach of trust” means when a party’s trust intention or belief decreases because of a trust policy being violated (Kim et al., 2009). A breach of trust may result in a variety of adverse effects, such as negative emotional, cognitive, and behavioral implications (Shu et al., 2021; van der Werff et al., 2023); acts of retaliation (Yan and Wu, 2016); a decreased sense of justice (Kennedy and Schweitzer, 2018); and the breakdown of bilateral cooperation (Bottom et al., 2002). This paper reviews the research in the field, considering the major effect that trustworthiness has on individuals’ ability to assess the truth of assertions.

Trustworthiness has been studied for decades, but they are mostly empirical studies (e.g., Poon, 2013; Lleó de Nalda et al., 2016; Reimann et al., 2022). The review literature is few, a relatively limited amount of research has been quantitatively analyzed. So, it is impossible to fully describe the status and trend of trustworthiness research. This makes it difficult for researchers to gain insights that apply to their specific research area and to effectively translate fragmented research findings into practice. Therefore, this study intends to utilize bibliometric methods to collate and summarize previous studies on trustworthiness. Meanwhile, CiteSpace software is used to analyze the trustworthiness literature which collects in the Web of Science’s core database over the past 10 years. With the aim of helping the researchers grasp changing trends and structures within relevant areas of research, references for an in-depth examination of the scenario that is currently in place and for cutting-edge dynamics and prospective trends in the field.

Bibliometrics is a branch of information science that has grown into one of the most active fields in the field of worldwide book intelligence. It reflects the trend of contemporary discipline quantification (Qiu et al., 2003). Quantitative analysis from the perspective of bibliometrics can summarize the development status of a research field more objectively. Nevertheless, by means of data mining, processing and measurement or mapping, CiteSpace can be used to graphically express knowledge frameworks, structures, interactions, intersections, derivations, and other internal connections (Liu et al., 2009). To guarantee the highest level of data display accuracy, the CiteSpace software has a sophisticated data processing system and strong visualization tools that include both structural and temporal indications. Researchers frequently use these indicators to carry out in-depth assessments and analyses of research frontiers and hotspots within domains, allowing them to promptly comprehend the most recent advancements and development patterns. One of the core functions of this software is detection and analysis of the research frontier and knowledge relationship (Jia et al., 2019), as a result, the CiteSpace knowledge graph has gained popularity in a variety of scientific fields for drafting literature reviews because of its benefits. By analyzing the status, hotspots and trends of trustworthiness research, researchers can benefit from the integrated and fragmented knowledge. The root causes and the latest development status can be understood. In addition, this way can also enrich trustworthy study contents and more easily apply researchers’ findings to areas of interest to support them in assessing new directions for future research.

It is worth noting that the majority of the papers are qualitative-based, and previous researchers have conducted quantitative studies in different ways around different topics of trustworthiness, such as using meta-analysis (e.g., Yang and Beatty, 2016; Travers et al., 2019; Siddique et al., 2022) to quantify the extent, breadth, and role of age-related confidence differences (Bailey and Leon, 2019) and to evaluate the role of almond nuclei in facial trustworthiness treatment (Santos et al., 2016). Propose a paradigm change to advise on developing trustworthiness through ethical public health practices (Best et al., 2021). Rely on 79 peer-reviewed quantitative empirical studies spanning more than two decades to demonstrate the complexity of trust in a global homeschool context (Shayo et al., 2021). Investigate trustworthiness among human machines (Song and Luximon, 2020), and research trustworthiness using rooted theoretical techniques (e.g., Cheer et al., 2015; Filieri, 2016).

However, several study themes in the field of trustworthiness research complicate existing research, making it difficult to adequately reflect the status quo, research hotspots, and evolutionary tendencies. The current literature lacks a comprehensive study strategy, resulting in significant variation in the operability of existing studies (Siuda et al., 2022), preventing meaningful comparisons of findings as well as sufficient quantitative analysis. To acquire a thorough grasp of the current state and growth of trustworthiness studies, as well as to diminish their subjectivity, we undertake a comprehensive assessment using knowledge graph analysis, with the goal of depicting the area comprehensively and methodically. The approach of literature metrology allows scholars to reflect the state and substance of trustworthiness studies, highlight the development trajectory of trustworthiness research, increase their grasp of the field’s evolution, and identify new directions more directly. On this basis, the study uses 1,463 pieces of relevant literature, quantitative literature measurement analysis, and trustworthiness-related studies to tackle the following research questions:

1. Which authors and journals are regularly referred to in trustworthiness studies, acting as a jumping–off point to find high–impact research in the field?
2. What is the volume distribution of trustworthiness by time and region?
3. What is the main field of trustworthiness research?
4. Which research areas indicate expectations for the future?

## 2 Methods

**Figure 1:**
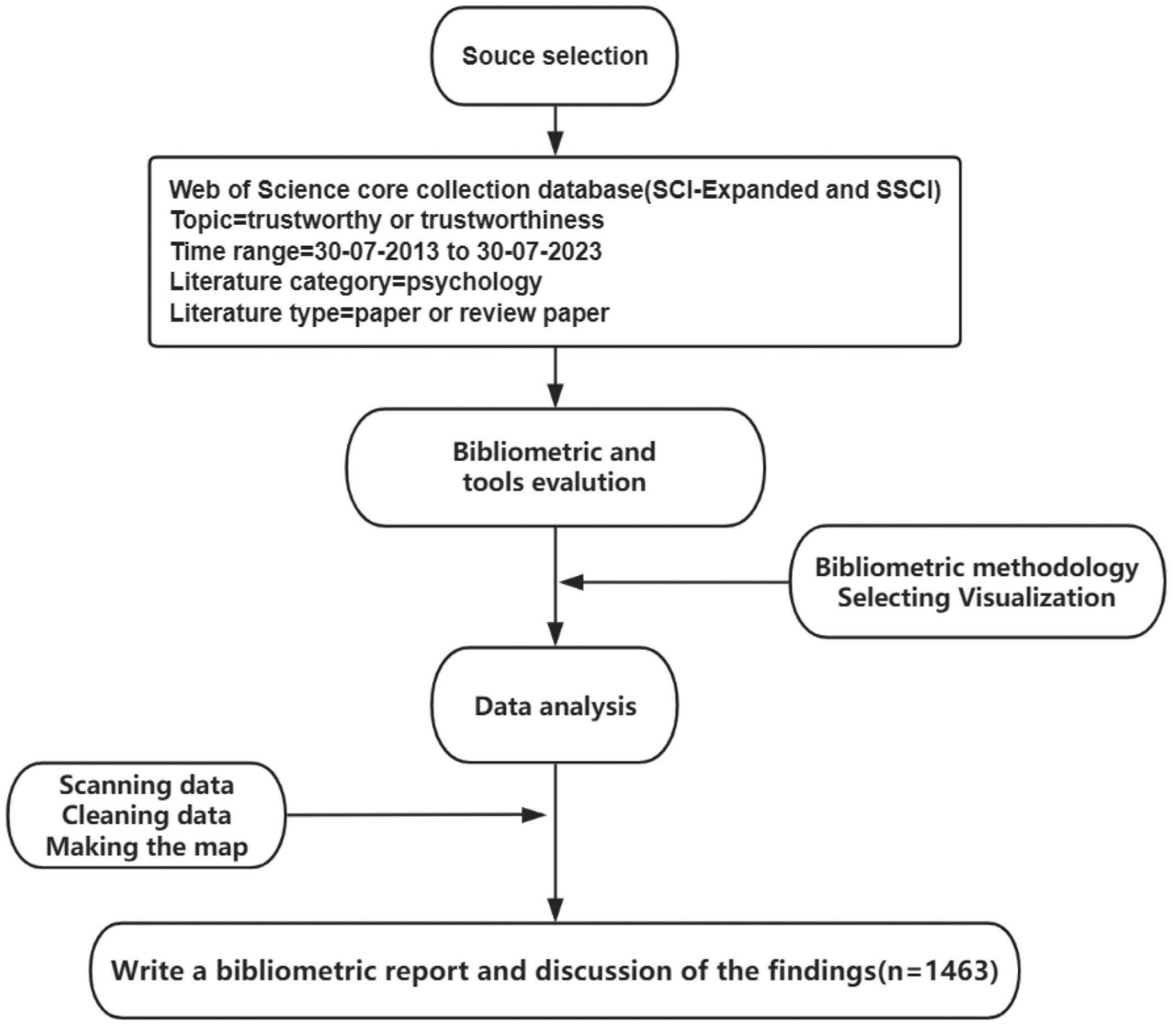
Process of making the map.

We set the thematic term to trustworthy or trustworthiness to collect effective and comprehensive objective literature. We applied certain limits before searching for subjects. First, we chose the Science Citation Index Expanded (SCI-Expanded) and Social Sciences Citation index (SSCI) from the Core database of the Web of Science as our research platform. It is the largest, complete database of academic information covering disciplines, with a wide range of time, quantity, and quality. Second, there have been fewer trustworthiness-related studies between the creation of the Web of Science database in 1985 and 2013, with a total of 24 pertinent pieces of literature that are not analytically reliable. The data from the past decade is more current and representative, providing a better reflection of the current academic field’s development trends and hotspots. Consequently, the relevant documentation published for 10 the past years was selected as the object of analysis, with the time range set to be from 30 July 2013 to 30 July 2023. Finally, we examine trustworthy as a psychological or perceptual value, referring to an individual’s or an organization’s characteristics or traits that inspire trust in them (Mayer et al., 1995). So, trustworthy in our study is a psychological trait, the literature type was set as article or review paper, and the literature category was limited to psychology, including multidisciplinary, social, applied, clinical, developmental, experimental, educational, biological, and mathematical psychology. A total of 1,463 studies were retrieved (see Figure 1).

The 1,463 works acquired by the study were visualized using an assortment of literature-determining and content-evaluation methods. First, the relevant documentation was obtained in pure text form from the Web of Science core collection database. Second, the visualization analysis software CiteSpace (6.2. R4) (hereinafter referred to as CiteSpace) was utilized to analyze the node type, which includes countries, authors, institutions, journals, and keywords. The time slice was set to 2 years. Third, we analyzed the results of the data analysis and relevant documentation, and the selected content was pruned by pathfinder to yield the corresponding knowledge maps.

## 3 Results

### 3.1 Spatiotemporal distribution

#### 3.1.1 Annual publication volume analysis

**Figure 2:**
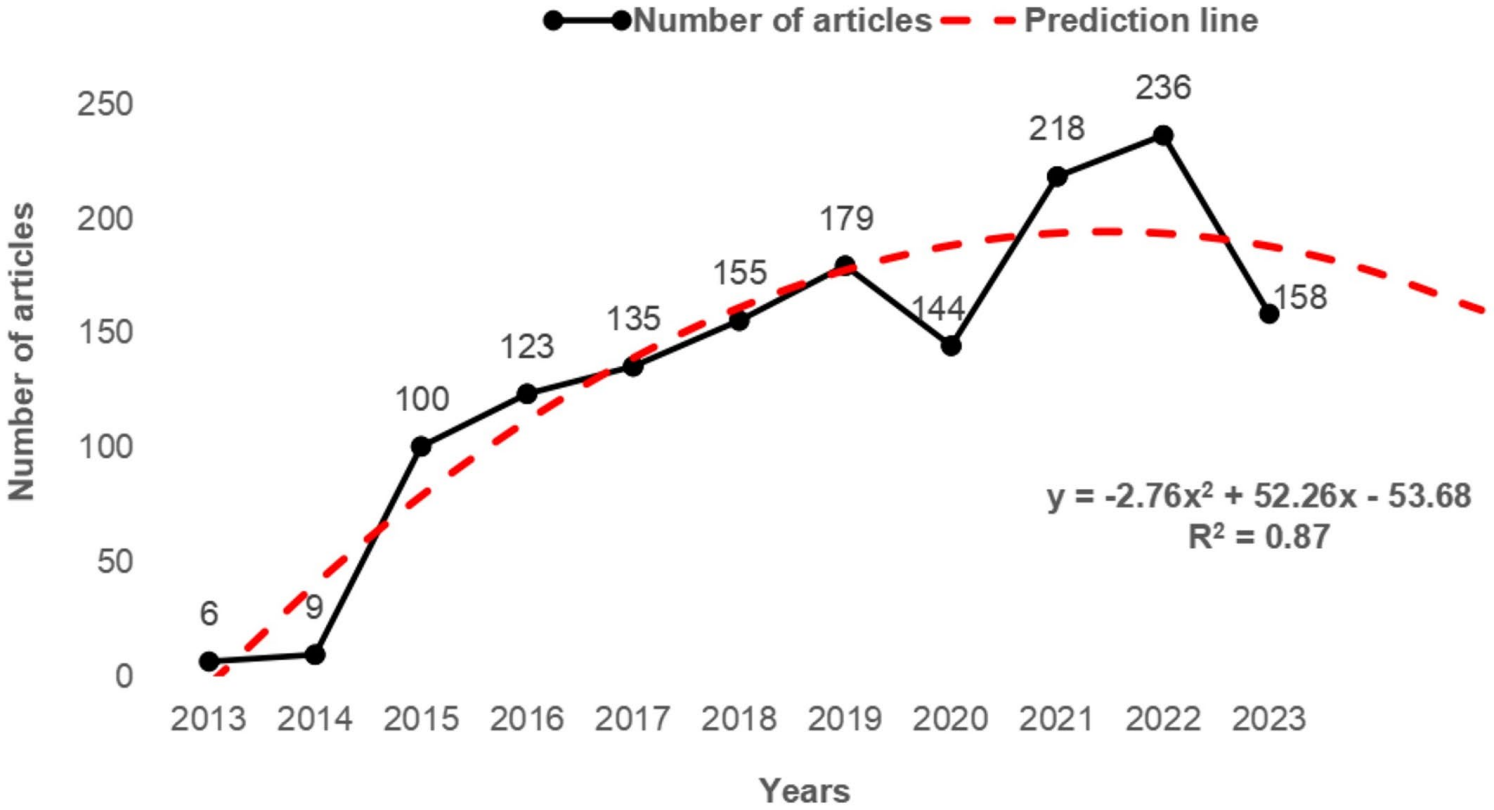
Annual publication volume.

The number of annual publications can reflect the development trend of a certain research field. Within the scope of retrieval, the annual amount of trustworthiness is shown in Figure 2, showing a rising trend. The development process in this field can be categorized into three stages: the phase of gradual advancement (pre-2014), the phase of rapid progression (2014–2015), and the phase of sustained growth (post-2015). We can discover that 2015 is a turning point in terms of items published by year. Before 2015, trustworthiness research reached a low level and continued in the enlightenment phase, this suggests that the study of trustworthiness is just beginning. After 2015, the volume of submissions significantly increased. Trustworthiness studies decline slightly in 2020 but then quickly recover and reach a new level in 2022 (236 articles). The publication of literature has varied over the past 10 years; instead, overall, the field of trustworthiness-related studies is moving forward. According to the polynomial fitting curve, trustworthiness studies are expected to remain at a more stable level for the next 2 years.

#### 3.1.2 Country analysis

**Figure 3:**
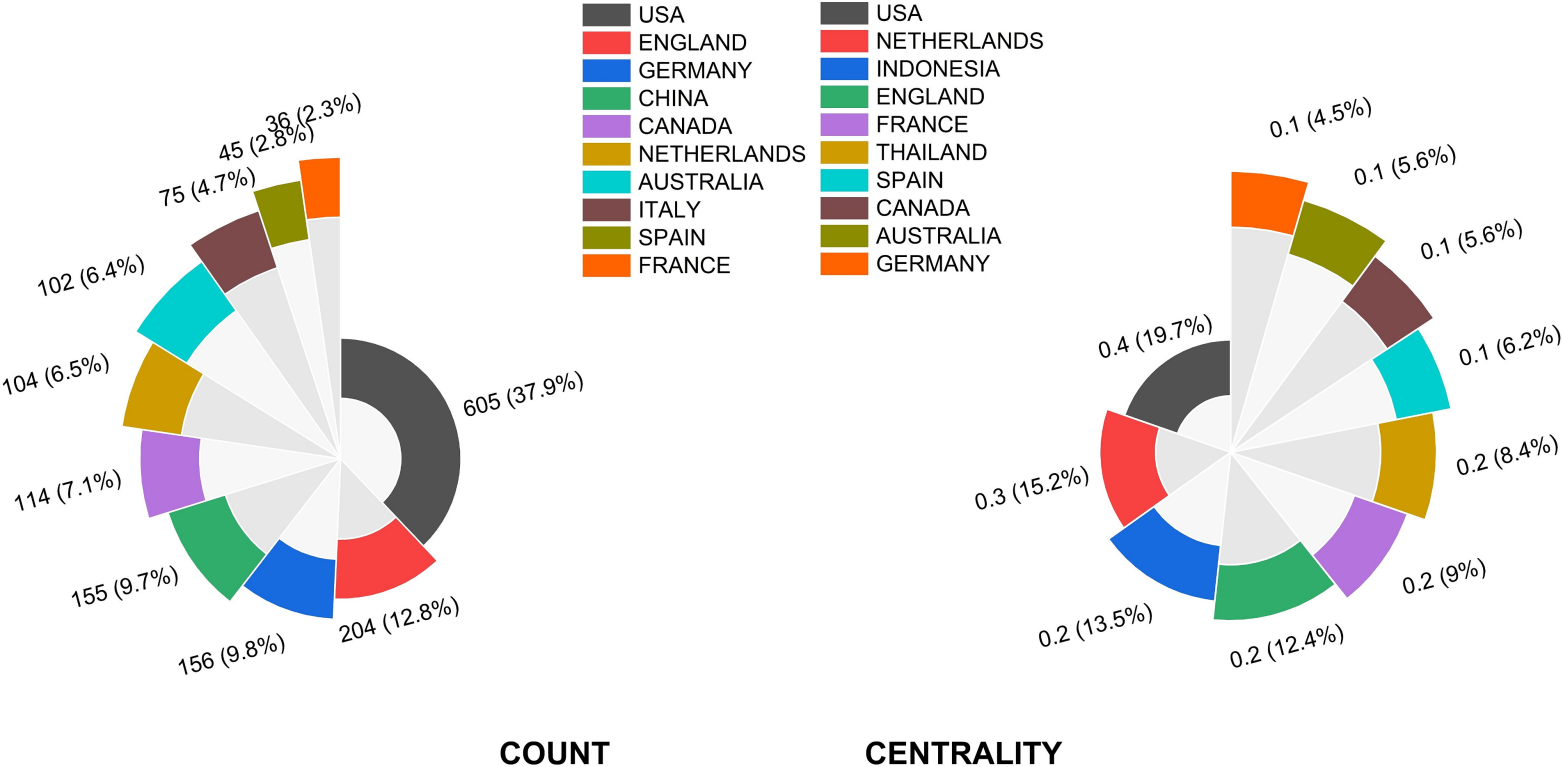
Chart of publication frequency of countries.

The top 10 countries in terms of articles and the value of trustworthiness research are presented in Figure 3. The United States has the most documents and the highest degree of centrality within the search area, indicating that it has the closest academic research relationship with other countries and contributed considerably to research innovation, which had a major effect. Despite the importance of trust, scholars have not given it much consideration for a long time. This hush did not break until the 1950s, when American psychologist M. Deutsch conducted the first experimental investigation of trust in the Prisoner’s Dilemma (Deutsch, 1958). Subsequently, many scholars in psychology, economics, sociology, political science, and other disciplines began to conduct in-depth research on trust issues from their own perspectives, resulting in an increase in trust research abroad and the formation of some relatively systematic trust research theories. This may be the reason why the United States is the highest degree of centrality country. The United Kingdom, Germany, China, Canada, the Netherlands, Australia, Italy, Spain, France, and other countries have high levels of production. The top three countries with the highest centrality are the United States, the Netherlands, and Indonesia. Germany and China publish the same number of papers, but their respective centralities are only 0.08 and 0.05, respectively, indicating that both have weak trustworthiness and should boost it.

### 3.2 Subject of publication

#### 3.2.1 Issuing institutions analysis

Table 1 lists the top 10 institutions based on count, centrality, and burst value. The top three universities in terms of volume are the University of California System, the University of London, and the N8 Research Partnership, and the top 10 research institutions in terms of publication volume published 354 articles. Harvard University, the University of California System, and the State University System of Florida are the top three universities, accounting for 24% of the total literature, with centrality values of 0.19, 0.15, and 0.15, respectively, all three universities are from the United States. The authors and journals together further confirm the important role and status of the United States in the field of trustworthiness research. The top three universities in terms of burst values are Yale University, the University of Toronto, and Northwestern University, with 4.17, 3.89, and 3.09, respectively.

**Table 1:** Institutions distribution by count, centrality and burst.

Institutions	Count	Institutions	Centrality	Institutions	Burst
University of California System	52	Harvard University	0.19	Yale University	4.17
University of London	49	University of California System	0.15	University of Toronto	3.89
N8 Research Partnership	43	State University System of Florida	0.15	Northwestern University	3.09
Harvard University	37	Columbia University	0.15	University of Oxford	3.04
White Rose University Consortium	34	University of York—UK	0.11	University of Cologne	2.70
University System of Ohio	31	University of London	0.10	Maastricht University	2.39
University of York—UK	29	University of California Los Angeles	0.10	Duke University	2.37
Tilburg University	27	University of North Carolina	0.09	Radboud University Nijmegen	2.36
University of Milano-Bicocca	26	University of Western Australia	0.09	University of Cambridge	2.33
State University System of Florida	26	CIVIS	0.09	Renmin University of China	2.26

#### 3.2.2 Cited journals analysis

**Figure 4:**
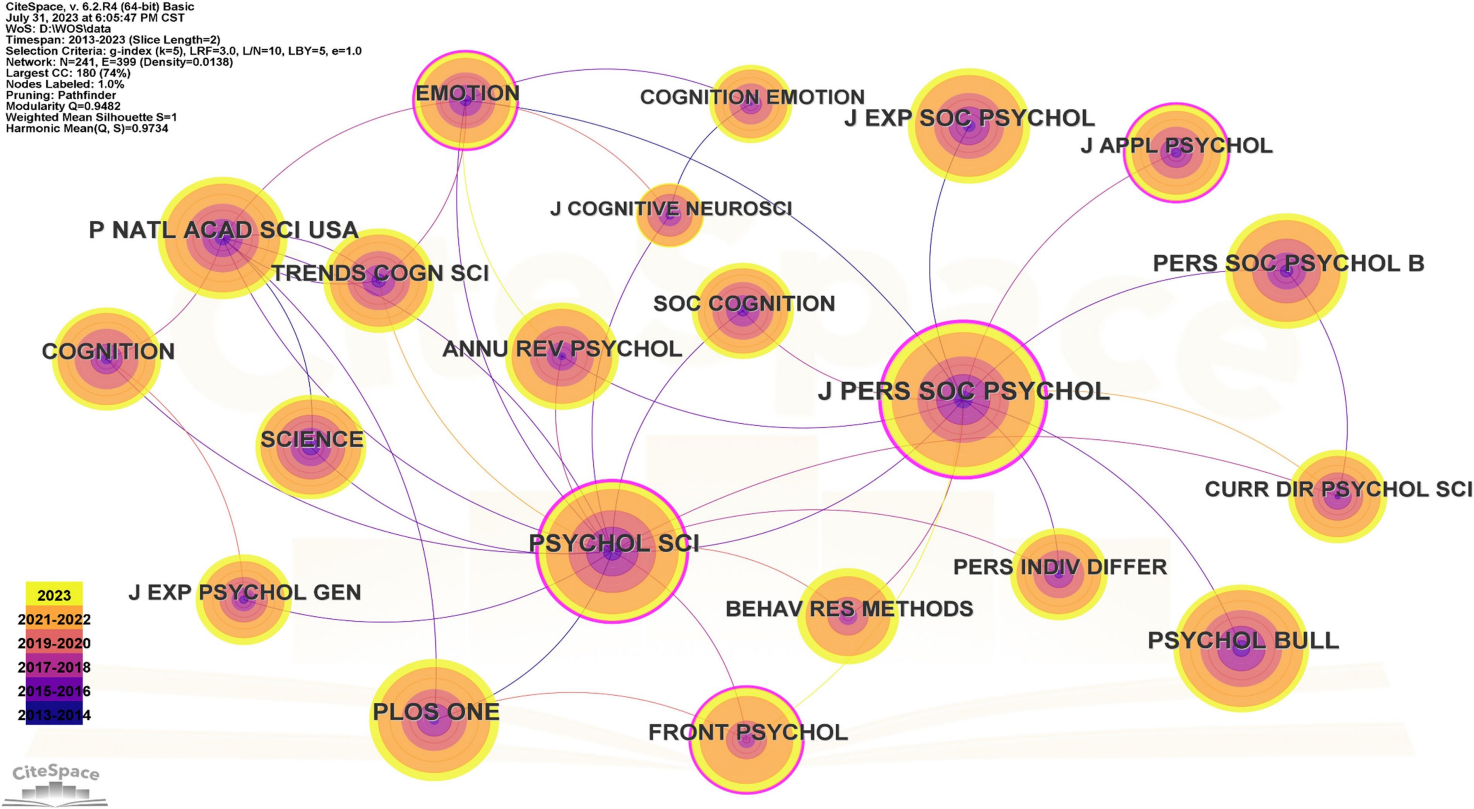
Cooperative network diagram of cited journals.

CiteSpace provides an illustration of the year and name of the cited journal with the size and color of the “Year Wheel.” The cited journal network knowledge graph involves a total of 241 nodes and 399 connections, with a density of 0.0138 within the criteria of the search (Figure 4). Evaluating the significant study results centered around trustworthiness becomes simpler through the analysis of these academic journals. As a result, Table 2 includes statistics for the 10 most common, centralized, and explosive publications. J Pers Soc Psychol was the most frequently cited journal (863 times), followed by Psychol Sci (724 times) and Psychol Bull (592 times). The journal with the highest centralization was J Pers Soc Psychol (0.43), followed by Psychol Sci (0.33) and J Appl Psychol (0.19). The most significant growth was in Biol Letters (12.27), followed by Thesis (9.50) and Nat Hum Behav (9.06).

**Table 2:** Distribution of cited journals.

Journals	Count	Journals	Centrality	Journals	Burst
J Pers Soc Psychol	863	J Pers Soc Psychol	0.43	Biol Letters	12.27
Psychol Sci	724	Psychol Sci	0.33	Thesis	9.50
Psychol Bull	592	J Appl Psychol	0.19	Nat Hum Behav	9.06
PLoS One	556	Organ Behav Hum Dec	0.16	Psychol Med	9.02
P Natl Acad Sci USA	555	Comput Hum Behav	0.16	J Vision	8.98
J Exp Soc Psychol	492	Front Psychol	0.15	Nat Commun	8.81
Pers Soc Psychol B	484	Acad Manage J	0.13	Soc Sci Med	8.65
Annu Rev. Psychol	431	Emotion	0.12	Cortex	8.28
Trends Cogn Sci	416	Cognition	0.08	J Educ Meas	8.04
Cognition	402	Neuropsychologia	0.08	J Conflict Resolut	7.79

Journal of Personality and Social Psychology mainly includes the empirical research reports related to personality and social psychology. Cottrell published an article entitled What do people desire in others? A sociofunctional perspective on the importance of different valued characteristics in this journal had been cited more than 211 times. The article states that trustworthiness is considered extremely important for all the interdependent others in different measures of trait importance and different groups and relationships (Cottrell et al., 2007). Biology Letters is a professional bio-journal published by Royal Soc Publisher. Rhodes et al. (2012) published an article titled Women can judge sexual unfaithfulness from unfamiliar men’s faces, which examines whether sexual trust (loyalty) can be accurately judged from the face of a stranger of the opposite sex. It concludes that for women, there is some evidence of judging sexual loyalty from their faces and further demonstrates that face perception appropriately adjusts to the signals of mate quality.

#### 3.2.3 Author analysis

**Figure 5:**
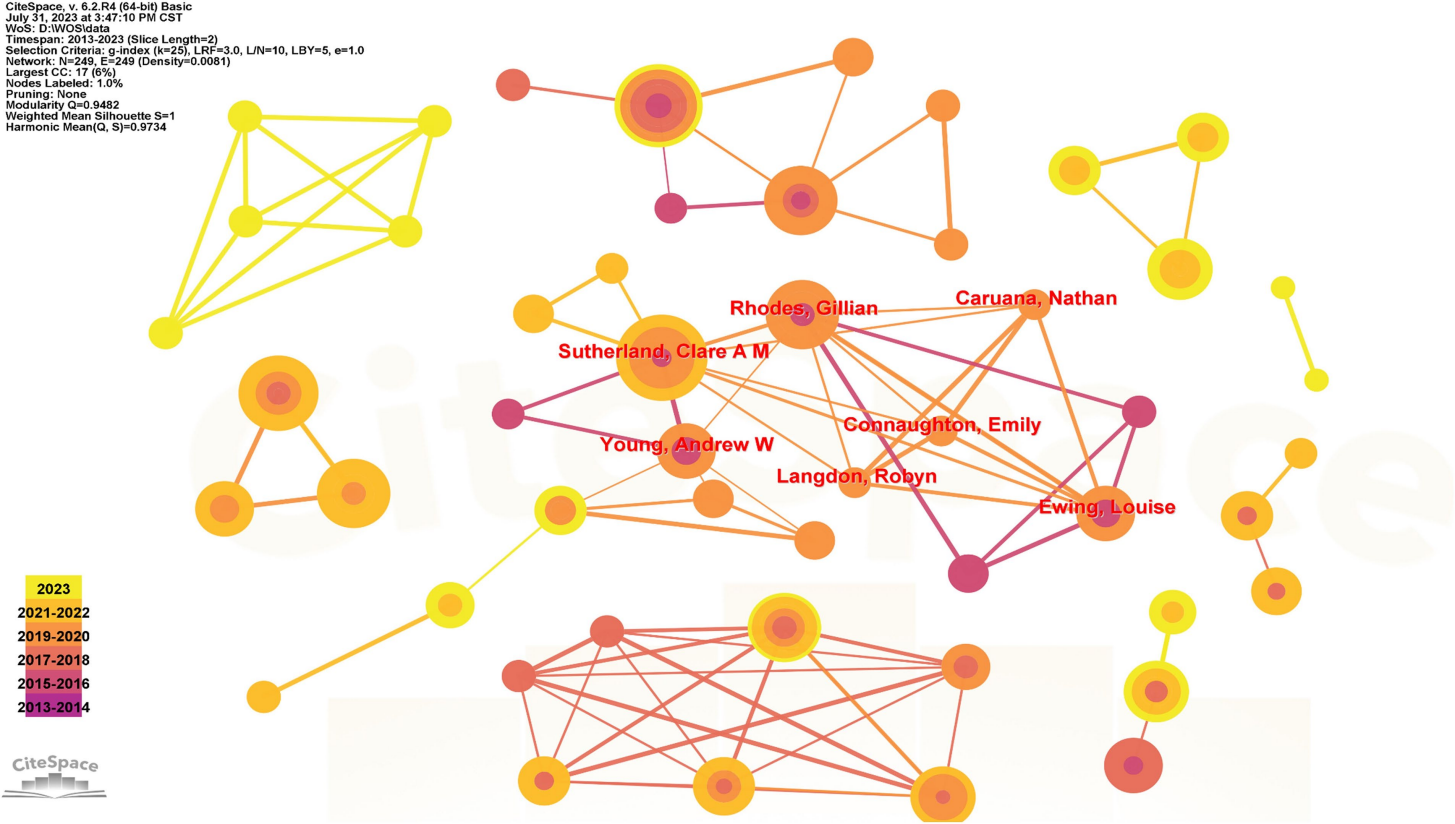
Author cooperation network diagram.

A network density of 0.0081, 249 nodes, and 249 connections are present. Only the authors with the highest links are shown in Figure 5. According to the figure, a crucial collaborative group has developed in the field of trustworthiness research with high-production authors like Sutherland, who primarily studies the detection of facial clues to trustworthiness. His most popular article is that social inferences from faces: ambient images generate a three-dimensional model, which developed and validated a 3D model (approachability, dominance and youthful-attractiveness) through 3 experiments, and studying two-dimensional valence or trustworthiness through a dominance model of face social inference. What’s more, his findings highlight both the utility of the original trustworthiness and dominance dimensions and the need to utilize various facial stimuli, as well as further highlight the importance of youth and attractiveness perception in facial assessment (Sutherland et al., 2013).

As leaders in the field of research, high-production authors or core authors not only control the field’s current research hotspots and directions but also influence the direction of subsequent research (see Tables 3, 4). Based on the number of submissions, the top three authors are Todorov A, Sutherland CAM, and Evans AM. The first three authors in burst value are Brambilla M, Tipper SP, and Rhodes G, with values of 3, 2.41, and 2.07, respectively. Brambilla M, Jaeger B, Masi M, and Mattavelli S are the most dynamic authors in the last 3 years when the number of citations is considered the number of citations of an article in other author references after publication. Todorov A, Oosterhof NN, and Willis J are the top three most cited authors. Jaeger B, Ma DS, and Glaeser EL are the top three most triggered authors, with values of 12.39, 10.22, and 8.96, respectively. The articles produced by the individuals mentioned above played a vital, pivotal, or revolutionary role in the research on trustworthiness.

**Table 3:** Authors distribution.

Authors	Count	Authors	Burst	Years (2013–2023)
Todorov A	15	Brambilla M	3	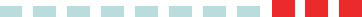
Sutherland CAM	14	Tipper SP	2.41	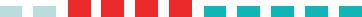
Evans AM	12	Rhodes G	2.07	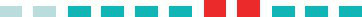
Dotsch R	10	Jaeger B	2.03	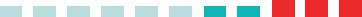
Rhodes G	9	Mieth L	2.03	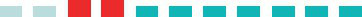
Jaeger B	9	Buchner A	2.03	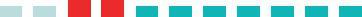
Alarcon GM	9	Topolinski S	1.98	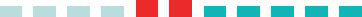
Brambilla M	8	Masi M	1.86	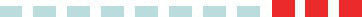
Over H	8	Mattavelli S	1.86	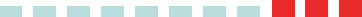
Lyons JB	8	Rule NO	1.81	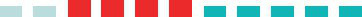

**Table 4:** Distribution of cited authors.

Authors	Count	Authors	Burst	Years (2013–2023)
Todorov A	415	Jaeger B	12.39	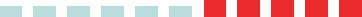
Oosterhof NN	279	Ma DS	10.22	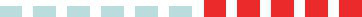
Willis J	234	Glaeser EL	8.96	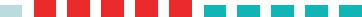
Mayer RC	214	Debruine LM	8.20	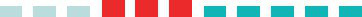
Zebrowitz LA	198	Eckel CC	7.24	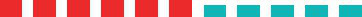
Fiske ST	185	Caulfield F	6.85	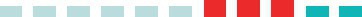
Rule NO	161	Delgado MR	6.58	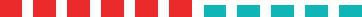
Faul F	157	Simpson JA	6.56	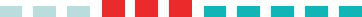
Berg J	153	Bayliss AP	6.28	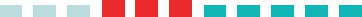
Colquitt JA	139	Suzuki A	6.25	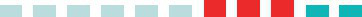

One of the authors who is frequently cited, Todorov A, also emphasized the trustworthiness of faces, linking them to emotions and neurons and studying how people perceive, evaluate, and absorb the social world. The author with the highest burst value, Brambilla M, also studied face trustworthiness. However, Brambilla showed that the facial aspect ratio and the tone of the voice can influence social perception. The research focused on the effect of auditory and visual cues on facial trustworthiness. The author Jaeger B, with the highest cited bursts, studied the effects of facial trustworthiness cues on social decision-making and social interactions. This indicates that research on trustworthiness focuses mainly on facial trustworthiness.

### 3.3 Thematic characteristics

#### 3.3.1 Keyword analysis

**Figure 6:**
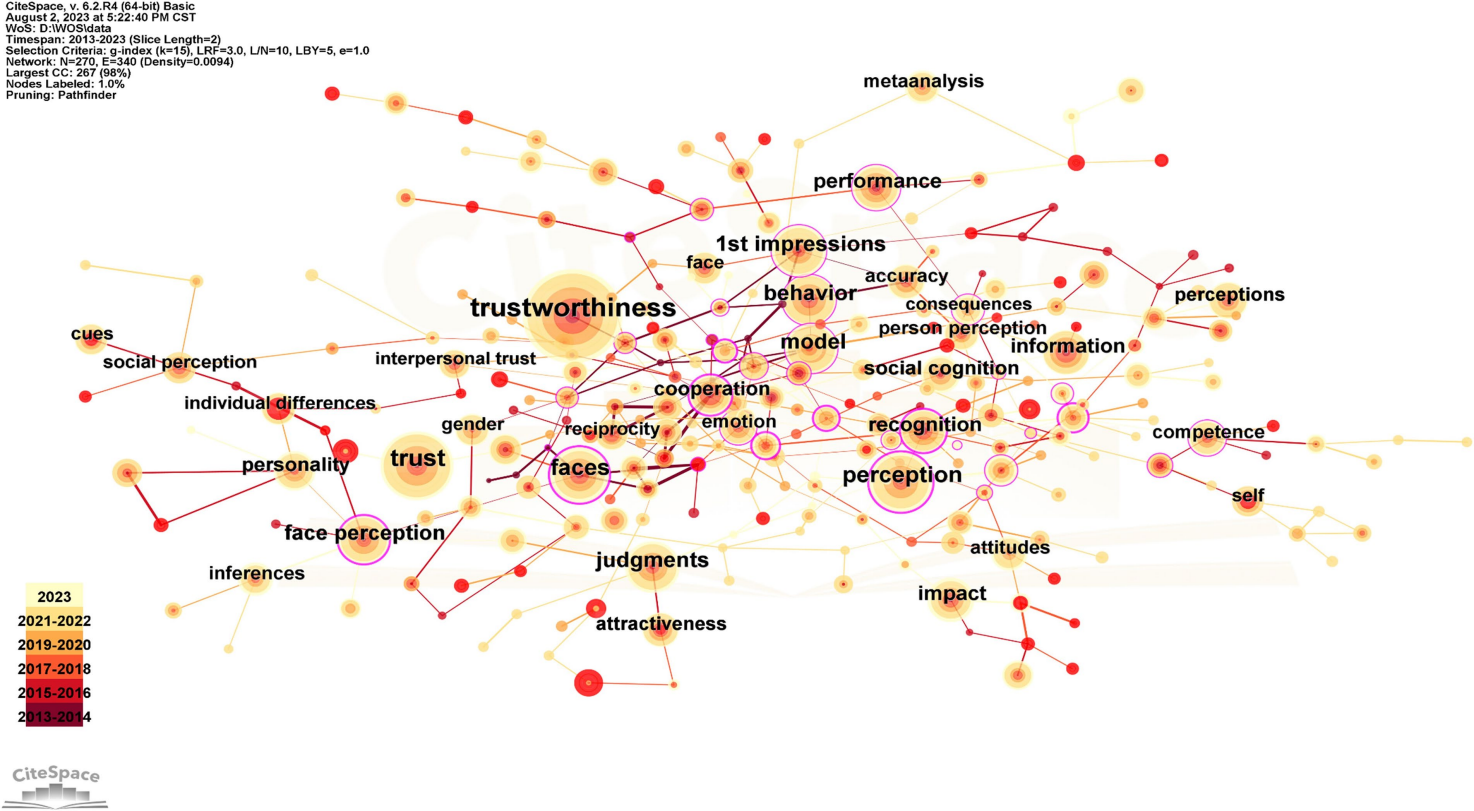
Keyword network diagram.

Keywords act as both an overview of the topic and the thesis’s main point of view. The amount of attention within an area of study can be seen by high keyword frequency and centralization. It may reflect advanced research methods, hot issues that need to be addressed, or academic topics that interest researchers over a given period. Within the scope of retrieval, there are 270 nodes and 340 links in the knowledge map of the keyword network, with a density of 0.0094 (see Figure 6). The top 10 keywords for frequency and centralization are listed in Table 5. The three keywords with the highest frequency are perception, trust, and trustworthiness, with frequencies of 387, 206, and 161, respectively. Competence, model, and facial expressions were the top three in terms of centrality, at 0.11, 0.10, and 0.09, respectively. Combining high-frequency keywords with associated literatures reveals that trustworthiness research focuses primarily on anex-dependent and post-dependent variables such as ability, facial expression, first impression, and So On. These keywords are closely related to other keywords around, which is more important and has greater influence in the research.

**Table 5:** Keyword distribution.

Keywords	Count	Keywords	Centrality
Trustworthiness	387	Competence	0.11
Trust	206	Model	0.10
Perception	161	Facial expressions	0.09
Faces	146	Faces	0.08
Judgments	125	Inferences	0.08
Behavior	123	Consequences	0.08
Model	122	Perspective	0.08
1st impressions	118	Children	0.08
Face perception	108	Age	0.08
Performance	93	Emotion	0.07

#### 3.3.2 Research frontier analysis

**Figure 7:**
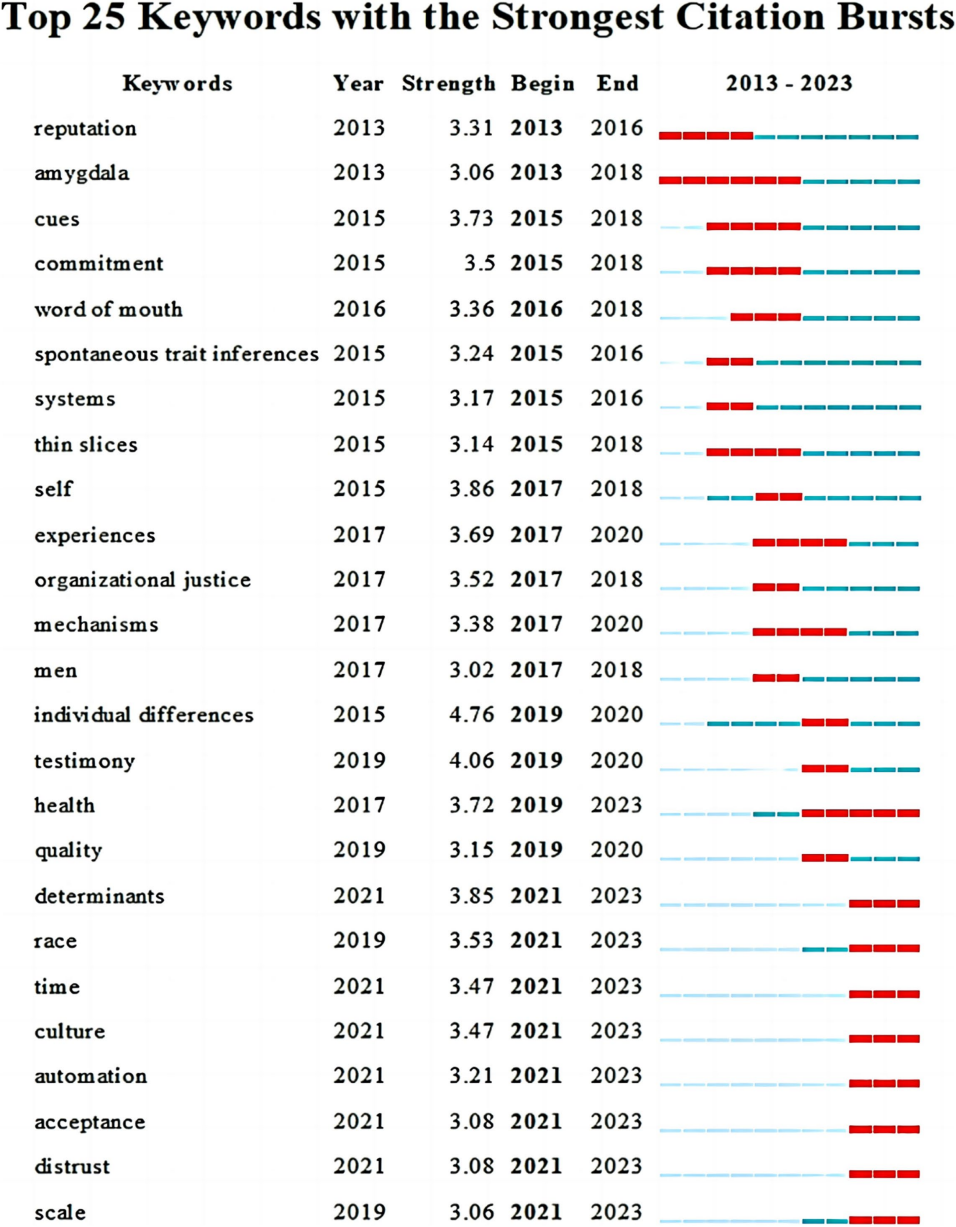
Keywords with the strongest citation bursts.

Burst words are words that change significantly in quoted frequency in a certain period. Through the analysis of hot words, they can reflect the hotspots and frontier dynamics in a certain research field. A list of burst words was generated for the timeline by using CiteSpace and selecting the top 25 burst words of the study, as shown in Figure 7.

Until 2016, reputation, spontaneous trait inferences, and systems had attracted more and more attention. At this time, trustworthiness research was driven by reputation systems (e.g., Kuwabara, 2015; Wibral, 2015; Pouryazdan et al., 2016; Wang et al., 2016) as well as spontaneity (e.g., Klapper et al., 2016). By the time 2018, these several key words get more attention, like amygdala, cues, commitment, word of mouth, thin slices, self, organizational justice and men. Furthermore, the trustworthiness of research at this time concentrated on connections between tactics like emotional (e.g., Caulfield et al., 2015), customary culture (e.g., Sofer et al., 2017), and commitment (e.g., Kam et al., 2016). By 2020, the focus of trustworthiness research is mainly on experiences, mechanisms, individual differences, testimony, and quality. For example, how the audience’s experience of sighting video in TV news affects the trustworthiness of reports (e.g., Halfmann et al., 2019); trust behavior and brain neurons (e.g., Wang et al., 2018; Zebrowitz et al., 2018). As of 2023, the current research focus has changed to health, determinants, race, time, culture, automation, acceptance, distrust, and scale. Trustworthiness research is not limited to the field of social communication, such as organizations, teachers, and students, but has gradually expanded to the medical field, for instance, health care, medical intelligence (Markus et al., 2021), and differences in trustworthiness between specific cultures or across cultures.

#### 3.3.3 Research topic analysis

**Figure 8:**
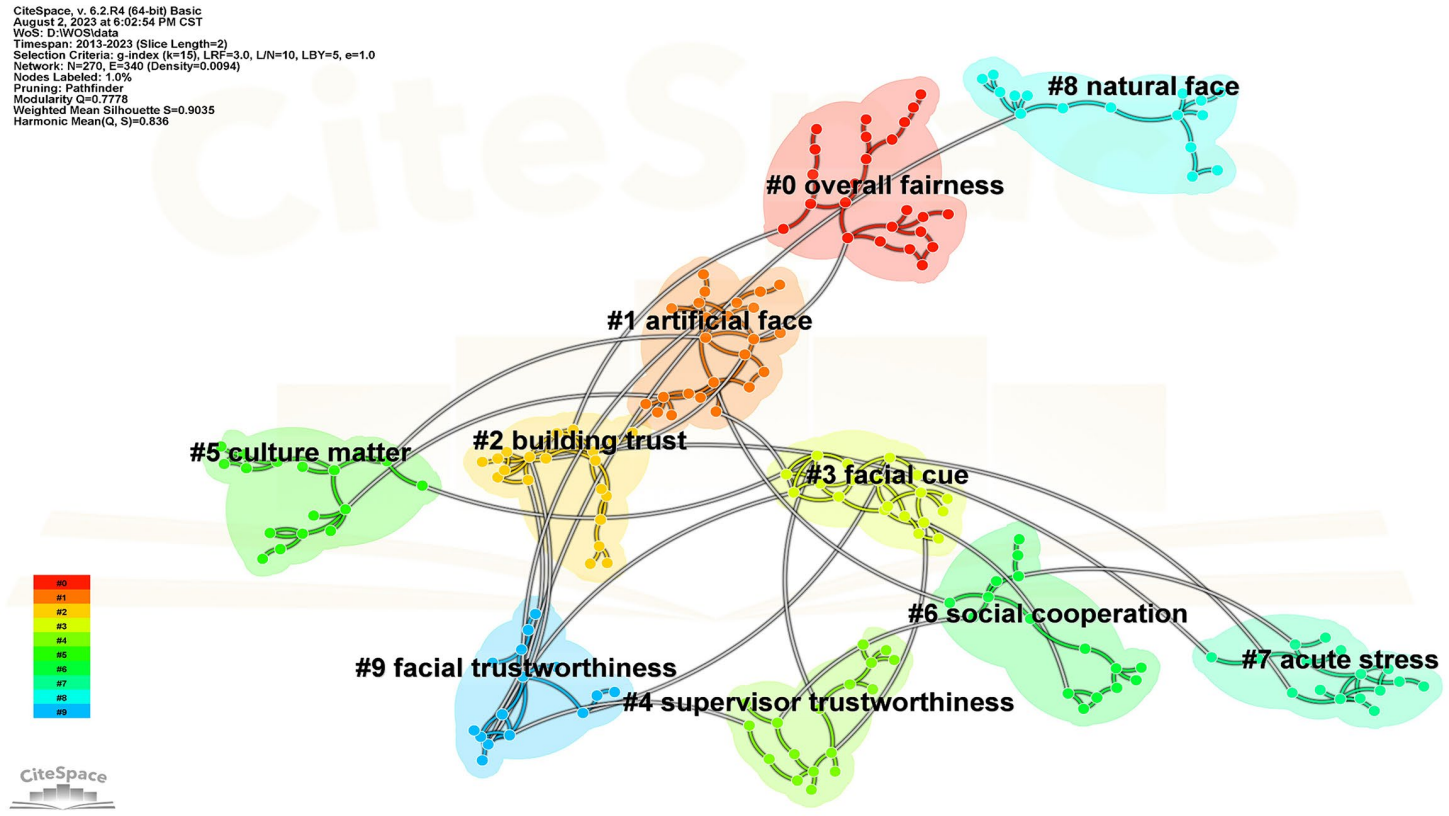
Keyword clustering network diagram.

The cluster analysis of keywords based on the keyword distribution network is shown in Figure 5, further reveals the topic of trustworthiness research. In Figure 8, the cluster modularization Q value is 0.7778 (Q > 0.30), indicating a substantial cluster network association structure. In addition, the average contour value (S) is 0.9035, indicating that the cluster results are real and may act as a trustworthy source of data for trustworthiness studies. Overall fairness (marks of 0), artificial face (marks of 1), building trust (marks of 2), and unique clustering information constitute the 10 keyword clusters that emerged (see Table 6). After summarizing and combining the research hot spots in this field using clustering graph and clustering label related indicators, it is discovered that the research hot spots exhibit “multiple diffusion,” which can be broadly classified into three core topics: facial cues, artificial intelligence, and social perception. This will be covered in depth in the discussion section.

**Table 6:** Keyword clustering information.

Cluster ID	Size	Silhouette	Mean (Year)	(Label) LLR
0	24	0.951	2017	Qualitative research; trust; artificial intelligence; justice; procedural justice
1	24	0.926	2017	First impressions; emotional expressions; facial trustworthiness; face recognition; artificial faces
2	20	0.899	2017	Face perception; risk; person perception; social preferences; trust
3	19	0.951	2015	Facial expression; emotion; smile; gaze cueing; happiness
4	17	0.915	2017	Person perception; social cognition; games; trait inferences; distrust
5	17	0.916	2018	Competence; warmth; social competence; cultural differences; stereotype
6	16	0.937	2016	Facial attractiveness; attractiveness; makeup; evolutionary psychology; physical attractiveness
7	16	0.796	2017	Psychology; others; economic games; halo effect; youth
8	16	0.952	2017	Face perception; social perception; open data; emotion recognition; open materials
9	15	0.901	2015	Evolution; expressions; thin slices; schizophrenia; continuous flash suppression

## 4 Discussion

Regarding positive expectations of the other party’s intentions and behavior, the extent to which one party is willing to take risks or expose itself to vulnerabilities is termed trust (Mayer et al., 1995). Trust is closely attached to interpersonal interactions such as reciprocity, cooperation, and betrayal (Lemmers-Jansen et al., 2017), and it serves an essential and dominant role in individual behavior (Falk and Hermle, 2018). People choose whether to act on trust based on perceived legitimacy; thus, trust does not just emerge out of thin air. Breuer and McDermott (2010) argue that trustworthiness is more important than trust for the success of public policy and sustainable long-term economic growth. In part because trustworthiness supports trust.

Most of the quantitative research on trustworthiness focus on a certain field, and our analysis has a basic understanding of the general framework of trustworthiness research through the citation knowledge graph. Most of the quantitative research on trustworthiness focus on a certain field, and our analysis has a basic understanding of the general framework of trustworthiness research through the citation knowledge graph. The results of this study show that the number of trustworthiness related studies has increased generally in the past decade; The trustworthiness research mainly focuses on the industrialized Europe and the United States, in which the research results of the United States have greater global influence; The University of California system, Harvard and Yale are among the most prolific institutions; The core authors are outstanding university scholars, represented by Alexander Todorov and others, but the level of cooperation among the core authors needs to be improved. The main journals that have published trustworthiness studies are *the Journal of Personality and Social Psychology* and *Biology Letters*. This report shows that cutting-edge research can be employed to divide trustworthiness-related research into three research directions: facial clues, artificial intelligence, and social perception. ABI model theory is a relatively popular and foundational theory for understanding trustworthiness, and although it was initially rooted in the context of trust within organizations, researchers have applied this model to a range of contexts, and there are many positive correlations between the three factors, so the ABI model is closely related to trustworthiness related research topics.

### 4.1 Facial cues and trustworthiness

Among the many factors that affect trustworthiness, facial clues have always been a hot topic of concern to researchers. Since the start of the 20th century, psychologists have known that there is general agreement that facial features are related to social and personality traits (Todorov et al., 2015). Face typicality is an important factor in social perception because it influences trustworthiness judgments. And the trustworthiness judgment is like the basic evaluation of the human face (Sofer et al., 2015). Wilson and Rule (2015) demonstrated how perceptions of people’s faces might be biased and influence their daily lives. According to Rhodes et al. (2012), facial indications have an early impact on trust behavior, and 10-year-olds preferentially trust partners they perceive to be trustworthy. The findings of Li et al. (2023) suggest that when one learns that another person is trustworthy (or unbelievable), the corresponding graphic traits in the mind are overlaid on the physical characteristics of the individual’s face. Then the facial characteristics are reshaped.

The current study also explores whether the perception of another person’s trustworthiness affects the characterization of the other person’s facial appearance and its potential mechanisms. At the same time, COVID-19 has made wearing masks common. When judging attractiveness, masks can enhance the attractiveness of less beautiful faces, but can reduce the attractiveness of more beautiful faces (Wang et al., 2023). Although one can form a stable first impression based on facial and vocal cues, their accuracy is low. Voice-based first impressions tend to be more positive than face-based first impressions (Jiang et al., 2024). In the study of facial trustworthiness, researchers have accumulated many theories (such as typical emotional generalization theory and typical theory) and experience. Future research on facial trustworthiness may be even deeper.

### 4.2 Artificial intelligence and trustworthiness

Intelligent technologies are increasingly entering the workplace. It has gradually shifted from workflow-supporting technologies to artificial intelligence (AI) agents as team members. And it has great potential in improving the health and well-being of the people (Ulfert et al., 2023). Although there are few applications for robots in clinical practice, they can benefit older people by reducing loneliness, troublesome behavior, and depression and improving social contact (Broadbent, 2017). Markus et al. (2021) discovered a lack of transparency as one of the major barriers to the clinical application of AI. They argue that explainable modeling can support reliable AI, though there was still an absence of useful evidence. But it may be used to support additional steps, such as reporting data quality, implementing extensive (external) validation, and regulation, to create trustworthy artificial intelligence. In the opinion of Song et al. (2023), regulatory compatibility expanded throughout par-social interaction and was a key element in the activation of social robot trustworthiness. To address the cognitive and emotional demands of users, artificial intelligence can also be used in the field of psychotherapy. It can simulate a variety of mental talents, including not just advanced processing and memory but also a few basic social and emotional abilities (Wiese et al., 2022).

Today, chatbot technology is constantly changing the interactive experience of traditional unguided online therapeutic intervention programs. It provides both human-like guidance and achieves full automation (Mo et al., 2023). However, researchers know very little about why chatbots operate, and there are currently no researchers to compile real, effective relationship clues to guide the design of chatbots. As a result, the investigation into the trustworthiness of AI may be in accordance with the evolving trends of new AI technologies.

### 4.3 Social perception and trustworthiness

The evaluation of trustworthiness of others included three aspects: ability, integrity, and benevolence, which could affect the perception of trustworthiness. When faced with integrity-benevolence and moral conflict, the individual’s trust behavior was also affected. For instance, Lupoli et al. (2020) demonstrated that while being viewed as having compassion can be a sign of goodwill, it does not always foster trust when presented with a moral dilemma. In addition, differences in trust and trustworthiness between cultures fell within the study. The study by Huang and Rau (2019) examined the impact of trust and trust in cooperation with friends or strangers in two different cultural business environments. The results revealed that Chinese and American participants had higher levels of trust and trustworthiness in friends than strangers. And Chinese participants were better able to distinguish between friends and strangers than American participants.

The degree of trust in both people and institutions is influenced by trustworthiness. Recent years have seen an increase in the frequency of emergencies, and how an organization responds to social emergencies has a bearing on its trustworthiness and the public’s level of trust in it. Emergencies are typically connected to institutions like governments. When people blame the government for environmental problems, their trust in the government declines (Kentmen, 2013). When negative events are not officially or authoritative, people are more faith in conspiracy theories (Xie et al., 2022). The result of liability attribution also has an impact on its relationship with the people (Ma and Zhan, 2016). Such research might strengthen the pillars of trustworthiness-related research further, opening the door for trustworthy applied research.

### 4.4 Analysis of the above three topics based on ABI model

Mayer and his colleagues conceptualize the trustworthiness structure as three interconnected factors: ability, benevolence, and integrity, which together determine whether a person or organization is trustworthy. Perceived ability is defined as the belief that a fiduciary can perform one or more specific tasks. Perceived benevolence is defined as the trustee’s perceived willingness to act in the best interests of the principal. Perceived integrity is defined as the degree to which the trustee’s values are believed to be compatible with their own. Mayer et al. (1995) present a complete theoretical framework for the concept of trustworthiness. Artificial intelligence, facial expressions, and social perception are all explainable using the ABI theoretical model.

Based on the ABI model’s trustworthiness and AI, Trust is a critical necessity for efficient human-computer interaction, as artificial organisms integrate into human civilization in a social setting. To fully integrate into our culture and optimize their acceptance and trustworthiness, artificial agents must adapt to the intricacies of their surroundings, just as people do. In a study of human-AI collaboration, indications of ability, warmth, and integrity influenced trustworthiness (Jorge et al., 2024). The use of artificial intelligence in psychotherapy necessitates replicating a wide range of psychological skills, including not only superior processing and memory but also some fundamental social and emotional capacities (Wiese et al., 2022). One of the key challenges to clinical AI application is a lack of openness in terms of data quality reporting, thorough (external) validation, and regulation to build trusted AI (Markus et al., 2021). Having advanced processing performance, social and emotional capabilities, and external oversight increases transparency, which corresponds to the three characteristics of trustworthiness.

Based on the ABI model’s trustworthiness and facial clues, many visual indicators, including facial expression and gender, influence people’s trustworthiness judgments at the same time. Entrepreneurs’ facial trustworthiness is positively correlated with the success of crowdfunding campaigns (Duan et al., 2020), and when a person’s facial expression conveys confidence and professionalism, others are more likely to believe that the person possesses the necessary skills and knowledge to complete the task. Happiness enhances the impression of trustworthiness, whereas anger diminishes it (Oosterhof and Todorov, 2009), and warm smiles, eye contact, and sympathetic expressions can also serve as indications of friendliness (e.g., Li et al., 2021). Angry facial expressions indicate immediate potential threats, and adults may predict violence and aggression based on face structure (Short et al., 2012).

Based on the ABI model’s trustworthiness and social perception. Ability, benevolence, and integrity influence trustworthiness in both individuals and organizations; yet, the definitions of trust and trustworthiness are sometimes implicit, and it may be unclear who or what is trusted. When trustees’ social identities increased, they were deemed more trustworthy (Xin and Zhang, 2018); prosocial liars are sometimes perceived as more trustworthy (Levine and Schweitzer, 2014).

## 5 Limitations and future research

### 5.1 Limitations

Although we conducted a topic search, so that the papers examined are the most relevant, The main drawback of co-citation analyses is the impossibility of fully collecting and displaying the entire existing literature (Stehmann, 2020). Firstly, only CiteSpace, a measurement analysis tool, and other readily available databases (Scopus, PubMed, etc.) and analytical tools (such as VOSviewer). Secondly, literature filtering duration is only 10 years and does not cover all relevant literature, were utilized in this study’s literature analysis of just one Web of Science database. Thirdly, the study is primarily based on empirical research and only from the realm of psychology, may not fully represent qualitative or transdisciplinary perspectives on trustworthiness. And lastly, the cited references list only the first authors instead of all authors, the citation rate does not reflect contributions of the second or further authors, which could affect citations accuracy regarding some authors (Garfield, 1979).

### 5.2 Future research

Since brain imaging technology has advanced, researchers have focused on the cognitive neural mechanisms underlying trustworthiness. They have discovered that, in addition to the almond nucleus, other brain regions-such as the internal frontal cortex and the right hip joint region—are also active during trust decision-making (Euston et al., 2023). Bellucci et al. (2019) combined a new paradigm for successfully inducing impressions of confidence through functional MRI and multivariate analysis. Studies have demonstrated integrity-based trustworthiness performance in the posterior cingulate cortex, dorsolateral prefrontal cortex, and intraparietal sulcus. Brain signals in these regions can predict the individual’s trust in subsequent social interactions with the same partner. Sijtsma et al. (2023) used functional magnetic resonance imaging (FMRI) to provide insights into how the behaviors and neural mechanisms of adolescent trust are affected by expectations. In the meantime, studies by Frazier et al. (2021) reinforced the idea that aging lessens sensitivity to traces of trustworthiness, but that intranasal oxytocin has no effect on behavioral adjustment.

In addition to FMRI techniques, event-related potential techniques (ERPs) have been further explored by using neural indicators reflecting electrophysiological activity in the cerebral cortex. P1, N17, early post-negative voltage (EPN), late positive component (LPC), and feedback negative waves (FN) have now been found to be important ERP indicators in this field (Leng et al., 2020). These findings grow our understanding of the neurological underpinnings of specific social traits. However, the accuracy and ecological usefulness of the results are negatively impacted by the measurement patterns and experimental materials, which are more uniform and primarily consist of static faces. Additionally, it is not clear which specific perceptual information—such as emotional cues or typicity—contributes more to brain region activation. With the development and combination of various technical means, this field can be discussed with more scientific and rigorous methods. In the future, researchers can continue to study the mechanism of the influence of trustworthiness on trust decisions through dynamic faces, to deeply explore the mechanism of the influence of trustworthiness on trust decisions.

In the case of trustworthiness-related empirical studies, it is typically divided between the trustor (the party whose trust has been violated) and the trustee (the one who carries out a trust violation). The relationship is clear and trust relationship is just one form of interpersonal relationships. However, the boundary between the responsibility subject and the responsibility is not so clear, especially in the collectivist environment like China. The situation may be more complex, and whether the research results can be extended to the real situation is worth further investigation.

Researchers have produced many experiences and results on trustworthiness studies. It is the focus of the field of economics and organization, and gradually radiated to the field of education. Brodsky et al. (2021) find that fact-checking strategies for improving college students through lateral reading teaching in general education civic courses require further research. Future research is needed to determine whether the improvement in lateral reading is maintained over time and to explore other factors. List and Oaxaca (2023) try to ascertain the effectiveness of college students participating in study report critique, examining the effects and potential future directions of student critical capacity development. The study of Su and Kong (2023) find that Chinese music students in English will encounter some severe challenges in the teaching language course (EMI) due to their limited English proficiency. Alves-Wold et al. (2023) studied the writing motivation of K-5 students. However, because of the lack of teacher behavioral perspectives and the main emphasis on higher education in these studies, future research may take a more important turn when combined with the findings of the hot spots and effective keyword analysis. For instance, the trustworthiness of educators with various cultural backgrounds and grade levels is studied under the update of education policy, and how to apply it in practice is considered, such as the update of the evaluation system and steps to improve trustworthiness.

## 6 Conclusion

This study demonstrates that the number of trustworthiness related studies has increased generally in the past decade; The University of California system, Harvard and Yale are among the most prolific institutions; The core authors represented by Alexander Todorov and others. The main journals that have published trustworthiness studies are *the Journal of Personality and Social Psychology* and *Biology Letters.* Popular topics include facial trustworthiness, brain neurology, medical trustworthiness, and cultural differences. Three factors inform the hot spot direction: facial clues, artificial intelligence, and social perception.

## Data availability statement

The raw data supporting the conclusions of this article will be made available by the authors, without undue reservation.

## Author contributions

ZZ: Funding acquisition, Methodology, Writing – review & editing. WD: Formal analysis, Methodology, Writing – original draft. YW: Formal analysis, Methodology, Writing – original draft. CQ: Conceptualization, Writing – review & editing.
